# Low birth weight and its associated factors

**DOI:** 10.31744/einstein_journal/2018AO4251

**Published:** 2018-10-30

**Authors:** Andreia Ielpo Magalhães Moreira, Paulo Roberto Moreira de Sousa, Flavio Sarno

**Affiliations:** 1 Instituto Israelita de Responsabilidade Social, Hospital Israelita Albert Einstein, São Paulo, SP, Brazil.; 2 Hospital Israelita Albert Einstein, São Paulo, SP, Brazil.

**Keywords:** Birth weight, Child health services, Pregnancy outcome, Birthing centers, Peso ao nascer, Serviços de saúde da criança, Resultado da gravidez, Centros de assistência à gravidez e ao parto

## Abstract

**Objective:**

To calculate the frequency and evaluate the factors associated with low birth weight.

**Methods:**

A retrospective study, with data from pregnant women who participated in the *Programa de Atenção* às *Gestantes do Programa Einstein na Comunidade de Paraisópolis,* between 2011 and 2014, and who returned for the postpartum evaluation of their newborns. Variables related to the pregnant woman, pregnancy, and newborn were evaluated. The outcome variable was low birth weight, defined as <2.5kg. The associations between the independent variables and low birth weight were assessed by χ^2^ and Mann-Whitney tests. Logistic regression models analyzed the combined effects of the independent variables on low birth weight.

**Results:**

Data of 794 pregnant women and their newborns (52.1% males) were analyzed. The age of pregnant women varied from 13 to 44 years (median of 24 years), and the majority reported being married or living in cohabitation (74.7%), and having between 9 to 11 years of schooling (53.4%). The proportion of low birth weight was 7.6% (newborn mean weight of 3.2kg) and, in multivariate analysis, presence of twinning, age group of the pregnant women (showing protection for low birth weight between ages ≥18 years and <35 years), and cesarean section were associated with low birth weight.

**Conclusion:**

The proportion of low birth weight was 7.6% and twining, age of the pregnant woman, and cesarean delivery were associated with the occurrence of low birth weight.

## RESUMO

**Objetivo:**

Calcular a frequência e avaliar os fatores associados ao baixo peso ao nascer.

**Métodos:**

Estudo retrospectivo, com os dados das gestantes que participaram do Programa de Atenção às Gestantes do Programa Einstein na Comunidade de Paraisópolis, entre 2011 e 2014, e que retornaram para realizar a avaliação de seus recém-nascidos após o parto. Foram avaliadas as variáveis relacionadas à gestante, à gestação e ao recém-nascido. A variável desfecho foi o baixo peso ao nascer, definido como <2,5kg. As associações entre as variáveis independentes e o baixo peso ao nascer foram avaliadas por meio dos testes χ^2^e de Mann-Whitney. Modelos de regressão logística analisaram os efeitos combinados das variáveis independentes no baixo peso ao nascer.

**Resultados:**

Foram analisados os dados de 794 gestantes e de seus recém-nascidos (52,1% do sexo masculino). A idade das gestantes variou de 13 a 44 anos (mediana de 24 anos) e a maioria referia ser casada ou estar em união estável (74,7%) e possuir entre 9 a 11 anos de estudo (53,4%). A proporção de baixo peso ao nascer foi de 7,6% (peso médio do recém-nascido de 3,2kg) e, na análise múltipla, a presença de gemelaridade, a faixa etária das gestantes (mostrando proteção para o baixo peso ao nascer nas idades ≥18 anos e <35 anos), e o parto cesárea se associaram com o baixo peso ao nascer.

**Conclusão:**

A proporção de baixo peso ao nascer foi de 7,6% e gemelaridade, idade da gestante e parto cesárea se associaram com a ocorrência de baixo peso ao nascer.

## INTRODUCTION

Low birth weight (LBW) is defined by World Health Organization as being lower than 2.5kg. It is estimated that 15 to 20% of newborns in the world present with LBW, which would represent more than 20 million births a year. Additionally, there are variations in the proportions of LBW among the regions, namely, 28% in South Asia, 13% in Sub-Saharan Africa, and 9% in Latin America.^(^ ^1^ ^)^

In Brazil, evaluation of data between 1996 and 2011 from the *Sistema de Informações sobre Nascidos Vivos* (SINASC) [Information System about Liveborns] showed 8.0% of LBW in the 26 capital cities and in Brasília; in that, the highest rates were found in the Southeastern (8.4%) and Southern (8.0%) regions, and the lowest, in the Northern (7.2%), Northeastern (7.6%), and Central Western (7.4%) regions.^(^ ^2^ ^)^

Low birth weight is an important public health problem, because it is associated with neonatal mortality. A systematic review of the literature up to 2011 and meta-analysis reported an odds ratio of 8.5 associated with neonatal mortality in full-term newborns (≥37 gestation weeks) with a birth weight <2.5kg.^(^ ^3^ ^)^ In Brazil, a cohort study about neonatal mortality between 2011 and 2012 also showed that LBW is one of the associated factors.^(^ ^4^ ^)^ Besides neonatal mortality, LBW is associated with some morbidities, such as asthma^(^ ^5^ ^)^ and hypertension.^(^ ^6^ ^)^

Therefore, efforts have been made to identify the factors associated with LBW. In the investigation with SINASC data, between 1996 and 2011, improvements in maternal schooling levels and coverage of prenatal care were associated with reduction in risk of LBW in all regions of Brazil.^(^ ^2^ ^)^ Yet, female newborns and a mother who smokes were factors associated with increased risk of LBW, in the *Pesquisa Nacional de Demografia e Saúde da Criança e da Mulher* (PNDS) of 2006 [National Demographics and Children’s and Women’s Health Survey] *.* ^(^ ^7^ ^)^

## OBJECTIVE

To calculate the frequency and evaluate the factors associated with low birth weight.

## METHODS

This is a retrospective study, with information obtained from spreadsheets with data from records of pregnant women who participated in the *Programa de Atenção* às *Gestantes* (PAG) [Pregnant Women Care Program] from the *Programa Einstein na Comunidade de Paraisópolis* (PECP) [Einstein Program in Paraisópolis Community], between 2011 and 2014, and who returned for an evaluation of their newborn after birth. The study was approved by the Research Ethics Committee of *Hospital Israelita Albert Einstein* , number 1.449.675, CAAE: 53647316.9.0000.0071.

The PAG promotes educational activities, with information about care in pregnancy and with the newborn, acting as a complementary to prenatal care service.

The variable outcome was LBW, defined as <2.5kg, and the independent variables were those related to the pregnant woman, and her past history and housing conditions, to the gestation, and to the newborn. Frequency distributions were verified by means of histograms and boxplots. The qualitative variables were described by absolute and relative frequencies, and the quantitative variables by their minimum and maximum values and medians, as they do not present with a normal frequency distribution. Due to the possible dependence that could occur in cases of the existence of the same pregnant woman participating in PAG/PECP in different years and/or twins, a random drawing was made in these situations, to choose which newborn would participate of the sample. This process was done using the software Excel 2010 and its random function, and the criterion to include the newborn in the analysis was the one that was attributed lower random value. The associations of the independent variables and LBW were evaluated by χ^2^ test for qualitative variables and Mann-Whitney test for quantitative or ordinal qualitative variables. The combined effects of factors associated with LBW were evaluated by logistic regression model, in which all independent variables were verified by a stepwise process in both directions; that is, including and excluding variables one by one, until reaching a model that contained only variables with a p value less than 5% in the final model. Also, we verified possible first-degree interactions between the independent variables. The results of the final multiple model were presented in odds ratios, followed by 95% confidence intervals and p values, with the level of significance set at 5%.

## RESULTS

During the period from January 2011 to November 2014, a total of 1,692 pregnant women were registered at PAG/PECP, and among these, 812 returned for the assessment of their newborns. Eighteen information were excluded related to newborns from the same mother and/or twins, with a total sample size of 794 participants.

The general proportion of LBW was 7.6%, and the newborn weight varied from 1.3 to 4.9kg, with a mean of 3.2kg (standard deviation of 0.5kg).

The age of the pregnant women ranged from 13 to 44 years (median of 24 years), and most reported being married or living in cohabitation (74.7%), did not work (57.3%), did not study (82.7%), and had between 9 and 11 years of schooling (53.4%). Most pregnant women lived in homes they owned (57.0%), with three to four [all-purpose] rooms (58.8%), which housed three or more residents (65.0%), with 1.3 resident per room. We noted no statistically significant associations of the variables analyzed, in the comparison between newborns with and without LBW ( Table 1 ).

**Table 1:** Characteristics of pregnant women and their housing, according to birth weight of newborns

Variables	Birth weight (kg)			

	Total	<2.5	≥2.5	p value
Age of pregnant woman, years	24.0 [13.0-44.0]	24.0 [14.0-44.0]	24.0 [13.0-43.0]	0.724*
Age group of pregnant woman, years				0.684*
13-17	140 (17.6)	16 (26.7)	124 (16.9)	
18-24	291 (36.6)	16 (26.7)	275 (37.5)	
25-29	179 (22.5)	13 (21.7)	166 (22.6)	
30-34	120 (15.1)	8 (13.3)	112 (15.3)	
>34	64 (8.1)	7 (11.7)	57 (7.8)	
Marital status				0.234^†^
Single or divorced	197 (25.3)	19 (31.7)	178 (24.7)	
Married or living in cohabitation	583 (74.7)	41 (68.3)	542 (75.3)	
Work				0.515^†^
No	446 (57.3)	32 (53.3)	414 (57.7)	
Yes	332 (42.7)	28 (46.7)	304 (42.3)	
Study				0.778^†^
No	640 (82.7)	48 (81.4)	592 (82.8)	
Yes	134 (17.3)	11 (18.6)	123 (17.2)	
Schooling, years	10.0 [0.0-16.0]	8.0 [0.0-15.0]	10.0 [0.0-16.0]	0.209*
Schooling group, years				0.088*
0-8	300 (39.6)	29 (51.8)	271 (38.7)	
9-11	404 (53.4)	23 (41.1)	381 (54.4)	
>11	53 (7.0)	4 (7.1)	49 (7.0)	
Housing				0.620^†^
Owner	442 (57.0)	36 (60.0)	406 (56.7)	
Not owner	334 (43.0)	24 (40.0)	310 (43.3)	
Number of rooms	4.0 [1.0-9.0]	4.0 [2.0-8.0]	4.0 [1.0-9.0]	0.885*
Range of rooms				0.530*
1-2	78 (10.1)	6 (10.2)	72 (10.1)	
3-4	456 (58.8)	32 (54.2)	424 (59.2)	
>4	241 (31.1)	21 (35.6)	220 (30.7)	
Number of residents	3.0 [1.0-14.0]	3.0 [1.0-9.0]	3.0 [1.0-14.0]	0.719*
Range of residents				0.714*
1-2	272 (35.0)	21 (35.6)	251 (35.0)	
3-4	363 (46.7)	29 (49.2)	334 (46.5)	
>4	142 (18.3)	9 (15.3)	133 (18.5)	
Number of residents/room	1.3 [0.3-4.0]	1.3 [0.5-3.0]	1.3 [0.3-4.0]	0.767*
Total	794 (100)	60 (7.6)	734 (92.4)	

Results expressed by n (%) and median [minimum value-maximum value]. * Mann-Whitney test; ^†^ χ^2^ test.

Most newborns were male (52.1%) and not twins (98.5%). Most pregnant women were primigravidae (51.6%), had not experienced any miscarriages (81.0%), had received prenatal care within the public services (93.6%), and had delivered vaginally or with forceps (59.0%). There was a significantly higher proportion of twins (15.0% *versus* 0.4%) and of cesarean sections (62.7% *versus* 39.2%) among the newborns with LBW ( Table 2 ).

**Table 2:** Characteristics of newborns, gestation and past history of pregnant women, according to birth weight of newborns

Variables	Birth weight (kg)			

	Total	<2.5	≥2.5	p value
Sex				0.649^†^
Female	304 (47.9)	24 (51.1)	280 (47.6)	
Male	331 (52.1)	23 (48.9)	308 (52.4)	
Twins				<0.001^†^
No	782 (98.5)	51 (85.0)	731 (99.6)	
Yes	12 (1.5)	9 (15.0)	3 (0.4)	
Number of gestations	1.0 [1.0-9.0]	1.5 [1.0-7.0]	1.0 [1.0-9.0]	0.983*
Gestations				0.958*
1	405 (51.6)	30 (50.0)	375 (51.7)	
2	221 (28.2)	20 (33.3)	201 (27.7)	
3	85 (10.8)	5 (8.3)	80 (11.0)	
≥4	74 (9.4)	5 (8.3)	69 (9.5)	
Number of miscarriages	0.0 [0.0-3.0]	0.0 [0.0-3.0]	0.0 [0.0-3.0]	0.775*
Miscarriages				0.834^†^
No (0)	636 (81.0)	48 (80.0)	588 (81.1)	
Yes (>0)	149 (19.0)	12 (20.0)	137 (18.9)	
Prenatal care				0.437^†^
Health insurance	48 (6.4)	5 (8.8)	43 (6.2)	
Public service	707 (93.6)	52 (91.2)	655 (93.8)	
Mode of delivery				<0.001^†^
Vaginal/forceps	465 (59.0)	22 (37.3)	443 (60.8)	
Cesarean section	323 (41.0)	37 (62.7)	286 (39.2)	
Total	794 (100)	60 (7.6)	734 (92.4)	

Results expressed by n (%) and median [minimum value-maximum value]. ^†^ χ^2^ test; * Mann-Whitney test.

As to the independent variables, no interaction remained significant in the final model. In this model, the significant factors were twinning, with an odds ratio of 42.5 for LBW; age group, showing protection for LBW in cases of pregnant women aged ≥18 years and <35 years; and mode of delivery, with an odds ratio of 2.3 for cesarean sections ( Table 3 ).

**Table 3:** Logistic model adjusted to odds ratio for low birth weight (n=788)

Variables	Odds ratio (95%CI)	p value
Twins		
No (reference)	1.0	
Sim	42.5 (11.6-203.8)	< 0.001
Age (years)		
<18 (reference)	1.00	
18-24	0.4 (0.2-0.8)	0.007
25-29	0.4 (0.1-0.8)	0.018
30-34	0.4 (0.1-1.0)	0.049
≥35	0.7 (0.3-1.9)	0.524
Mode of delivery		
Vaginal/forceps (reference)	1.0	
Cesarean section	2.3 (1.3-4.2)	0.007

95%CI: 95% confidence interval.

## DISCUSSION

The general profile the sample comprised women with a median age of 24 years, most of them were married or in living in cohabitation, and with 9 to 11 years of schooling. In Brazil, the largest rates of LBW are in the Southeastern and Southern regions.^(^ ^2^ ^,^ ^7^ ^)^ Thus, to compare our results with data from the Brazilian literature, we chose to evaluate the studies carried out in these regions of the country, since the pregnant women and the newborns of our sample belonged to the neighborhood of Paraisópolis, located in the city of São Paulo (SP). Nevertheless, the different dates, locations and samples evaluated, which could have influenced the characteristics of research participants, should be taken into consideration. One should also consider that the outcome variable was birth weight, regardless of the presence or not of prematurity, or of the evaluation of the newborn weight relative to the gestational age.

The proportion of LBW observed (7.6%) is below that reported in literature. In the city of São Paulo (SP), between 2007 and 2013, the rates of LBW varied from 9.6% to 9.8%;^(^ ^8^ ^)^ in the city of Divinópolis (MG), it ranged from 8.9% to 9.2%, between 2008 to 2011;^(^ ^9^ ^)^ and in the city of Taubaté (SP), between 2006 and 2010, it varied from 9.3% to 9.8%.^(^ ^10^ ^)^ In these comparisons, one should take into account that in the last two studies, twin birth were excluded from analyses, which could have influenced the proportions of LBW.

Regarding the characteristics of newborns, our study showed a mean weight similar to that of the study conducted in the city of Divinópolis (MG) (3.2kg *versus* 3.1kg),^(^ ^9^ ^)^ and a smaller proportion of females relative to the study done in the city of São Paulo (SP) (47.9% *versus* 51.2%).^(^ ^8^ ^)^ It is worth mentioning that we only evaluated newborns that returned to the program; non-return could have been due to birth or health conditions of these newborns, among other reasons, which could have influenced both their characteristics and the LBW rates.

In the comparison with the study carried out in the city of São Paulo (SP),^(^ ^8^ ^)^ the pregnant women of this sample were younger (24.0 years *versus* 27.5 years), had less cesarean sections (41.0% *versus* 56.5%), and a similar proportion of single gestations (98.5% *versus* 97.3%). Relative to the study on the spatial distribution of liveborns in the same city in 2008, our sample showed smaller proportions of pregnant women with up to 11 years of schooling (53.4% *versus* 58.2%), and similar extent of primigravidae (51.6% *versus* 53.9%).^(^ ^11^ ^)^ We should consider, however, that participation of the pregnant women in the program was spontaneous, and they belong only to one neighborhood in the city of São Paulo.

There was an association between age of the pregnant woman and the chance of LBW, a result that is in accordance with the literature. A systematic review with meta-analysis of the studies conducted in Latin America, up to 2008, showed that maternal age (<20 years and >35 years) was a risk factor for LBW.^(^ ^12^ ^)^ However, we should remember that socioeconomic factors can influence the risk of LBW associated with maternal age.^(^ ^13^ ^)^ Despite this, a systematic review of the literature on the complications of pregnancy in adolescence demonstrated this age group would present a higher frequency of other maternal and neonatal complications.^(^ ^14^ ^)^

As to twinning, the association with LBW has been noted in other studies, such as the one conducted in Botucatu (SP), between 2004 and 2008, with an odds ratio of 20.0 in twin pregnancies,^(^ ^15^ ^)^ and in Campinas (SP), in 2001, where double and triple pregnancies of full-term newborns presented with odds ratios of 19.9 and 21.4 for LBW, respectively.^(^ ^16^ ^)^

The association of cesarean delivery and LBW observed here has also been reported in the literature. In the State of Rio Grande do Sul, cesarean deliveries represented a risk for LBW, with an odds ratio of 1.1 among single newborns.^(^ ^17^ ^)^ However, one should bear in mind that this association is complex, since the procedure can be indicated in clinical or obstetric conditions related to complications for the mother or the fetus,^(^ ^18^ ^)^ and such conditions could be associated with LBW newborns. Additionally, the association between LBW and cesarean section might depend on other factors, such as the procedure rate.^(^ ^19^ ^)^

Several other variables have been identified in the literature as being associated with LBW; the most often cited are age and schooling level of the mother, number of prenatal care visits, sex of the neonate, and duration of gestation.^(^ ^20^ ^)^ As to maternal nutrition, a study with puerperal women of the city of Rio de Janeiro (RJ) showed that pre-gestational low weight was a risk factor, and weight gain during the gestation was a protection factor for LBW.^(^ ^21^ ^)^ In addition to these factors, the regional differences in the proportions of LBW in Brazil showed an association with the indicators that reflected the availability of the perinatal care services, socioeconomic factors, infant mortality rate,^(^ ^19^ ^,^ ^22^ ^)^ multiple births and newborns with very low weight, and neonatal mortality rate.^(^ ^23^ ^)^ In this way, one can perceive that the occurrence of LBW is multifactoral and involves maternal, newborn, gestational, and local context factors, among others.

## CONCLUSION

Our study presented the frequencies of low birth weight, and the characteristics of the pregnant women and their past history, housing conditions, gestations, and newborns. The variables twinning, age of the pregnant woman, showing protection for the newborns of pregnant women aged ≥18 years and <35 years, and cesarean section births were associated with the occurrence of low birth weight.
